# A Phase 1 trial of autologous monocytes stimulated ex vivo with Sylatron^®^ (Peginterferon alfa-2b) and Actimmune^®^ (Interferon gamma-1b) for intra-peritoneal administration in recurrent ovarian cancer

**DOI:** 10.1186/s12967-018-1569-5

**Published:** 2018-07-16

**Authors:** Daniel S. Green, Ana T. Nunes, Virginia David-Ocampo, Irene B. Ekwede, Nicole D. Houston, Steven L. Highfill, Hanh Khuu, David F. Stroncek, Seth M. Steinberg, Kathryn C. Zoon, Christina M. Annunziata

**Affiliations:** 1 Women’s Malignancies Branch, Center for Cancer Research, National Cancer Institute, National Institutes of Health, 10 Center Drive RM 3B43C, Bethesda, MD 20892 USA; 2 Cell Processing Section, Department of Transfusion Medicine, Clinical Center, National Institutes of Health, Bethesda, USA; 3 Biostatistics and Data Management Section, National Cancer Institute, National Institutes of Health, Bethesda, MD USA; 4 Laboratory of Infectious Diseases, National Institutes of Allergy and Infectious Diseases, National Institutes of Health Bethesda, Bethesda, MD USA; 5 Present Address: Office of Tissues and Advanced Therapies, Center for Biologics Evaluation and Research, FDA, Silver Spring, MD USA

**Keywords:** Cellular therapy, Immunotherapy, Monocytes, Interferons, Ovarian cancer, Intraperitoneal

## Abstract

**Background:**

Ovarian cancer has no definitive second line therapeutic options, and largely recurs in the peritoneal cavity. Locoregional immune therapy using both interferons and monocytes can be used as a novel approach. Interferons have both cytostatic and cytotoxic properties, while monocytes stimulated with interferons have potent cytotoxic properties. Due to the highly immune suppressive properties of ovarian cancer, ex vivo stimulation of autologous patient monocytes with interferons and infusion of all three agents intraperitoneally (IP) can provide a strong pro-inflammatory environment at the site of disease to kill malignant cells.

**Methods:**

Patient monocytes are isolated through counterflow elutriation and stimulated ex vivo with interferons and infused IP through a semi-permanent catheter. We have designed a standard 3 + 3 dose escalation study to explore the highest tolerated dose of interferons and monocytes infused IP in patients with chemotherapy resistant ovarian cancer. Secondary outcome measurements of changes in the peripheral blood immune compartment and plasma cytokines will be studied for correlations of response.

**Discussion:**

We have developed a novel immunotherapy focused on the innate immune system for the treatment of ovarian cancer. We have combined the use of autologous monocytes and interferons alpha and gamma for local–regional administration directly into the peritoneal cavity. This therapy is highly unique in that it is the first study of its type using only components of the innate immune system for the locoregional delivery consisting of autologous monocytes and dual interferons alpha and gamma.

*Trial Registration* ClinicalTrials.gov Identifier: NCT02948426, registered on October 28, 2016. https://clinicaltrials.gov/ct2/show/NCT02948426

## Background

Ovarian cancer is the leading cause of death due to gynecological malignancies, and the fifth leading cause of death due to cancer in women. Standard of care for the treatment of ovarian cancer is tumor cytoreductive surgery followed by administration of platinum and taxane based chemotherapy [1]. The disease course is characterized by a high rate of relapse despite an initial good response to the therapy [2]. Without curative second line treatment for patients with resistant or refractory epithelial ovarian cancer (EOC), median survival is 16 months, with most of the population dying within the first 2 years [3].

Despite the aggressive nature of ovarian cancer, and its high mortality rate, the disease is largely retained to the peritoneal cavity, with metastatic seeding to all of the major organs of the peritoneal cavity. Distant metastases (lung, brain) are infrequently found, and typically occur late in the course of disease. In most women, mortality is associated with abdominal disease [4]. The presence of the bulk of disease in the peritoneal cavity, and the semi-permeable nature of the peritoneum, makes ovarian cancer an ideal candidate for the use of locoregional therapy.

In 2006 there was a major advance in the treatment of some women diagnosed with EOC [5]. Patients with stage III optimally resected cancer were given cisplatin intraperitoneal (IP) and paclitaxel intravenous (IV) and IP, compared to standard IV administration of both drugs. The IP regimen resulted in a 15-month increase in overall survival compared to the standard IV therapy. This study showed that IP delivery of agents was a viable therapeutic option, and that IP therapy could increase efficacy of treatment. Recently it was shown that hyperthermic intraperitoneal chemotherapy (HIPEC) given at the time of surgery resulted in longer recurrence free survival and overall survival when compared to surgery alone. This study reinforced the observation that locoregional therapy augments systemic therapy.

The immune system is characterized by the innate immune system and the adaptive immune system. While these definitions were based largely on the host response to infection, they can be applied to the role of the immune system in cancer. Innate immunity is defined by a broad, non-specific, inflammatory process mediated by innate immune cells such as monocytes, natural killer cells and macrophages, and soluble mediators such as lipids and cytokines. The innate immune response also primes the adaptive immune response. Adaptive immunity is characterized by a highly specific response to an antigen or antigens expressed by an infectious agent or a cancer cell. Studies have shown that both the innate and adaptive immune system are important in the immune response to neoplasms. The majority of current cancer immunotherapies are targeting molecules and cells of the adaptive immune response either through agonist monoclonal antibodies or T cell therapies.

In the past several years there has been a massive expansion of the use of cell based immunotherapy for the treatment of metastatic cancer [6, 7]. The four major types of cell based immunotherapy are dendritic cell based vaccines, expanded tumor infiltrating lymphocytes, re-programmed T cells transduced with a cancer antigen specific T cell receptor gene, and chimeric antigen receptor (CAR-T) transduced T cells. Despite the success of CAR-T cells in blood malignancies, they have had limited and sporadic effects. One of the potential limitations of CAR-T therapy is the ability to deliver the cells into the tumor. An ongoing clinical trial for women with ovarian cancer, using a CAR-T directed against the ovarian cancer tumor antigen MUC-16, is exploiting the natural history of the disease by infusing the cells both IP and IV [8].

While the adaptive immune system has been the focus of most immune cell based therapies, some studies have suggested that innate immune modifiers can induce a clinical response. Interferons comprise a class of proteins that have potent anti-viral, anti-fungal, antibacterial and immunomodulatory properties. Furthermore, IFNs have been shown to be both cytostatic and cytotoxic to cancer cells, in vitro and in vivo [9]. During the late 1980s and 1990s, Phase 1 trials were completed testing IP infusion of immune modifying agents, including, but not limited to IFN-α or IFN-γ for treatment of cancers involving organs in the peritoneal cavity. Studies focused on, but were not limited to, the treatment of ovarian cancer. In order to support translating these findings to the clinic, we showed that monocytes from healthy donors have potent cytotoxic properties against a number of cancer cell lines when incubated with IFNs [10]. We focused our work on ovarian cancer due to the ability to deliver the therapy IP.

### Phase I clinical studies

The large-scale manufacture of cytokines and the production of therapeutic grade leukocytes through counter-elutriation paved the way for infusion of immune cells and cytokines, for the treatment of malignancies of the peritoneal cavity. The first Phase 1 study of IP immunotherapy for ovarian cancer evaluated infusion of high dose IFNα-2a as salvage therapy for patients with high grade epithelial ovarian cancer. Of 14 patients receiving IFNα-2a over 16 weeks, 6 patients (43%) had complete response, 1 patient (7%) had partial response, and 6 patients (43%) had disease progression [11, 12]. There was found to be an increase in NK cell activity and antibody dependent cell cytotoxic activity in response to IFNα-2a in 11 of the patients that were treated [13].That trial was followed by a study of 20 patients treated for 8 weeks. Eleven patients (55%) responded, and five of those patients (25%) had complete response. The median duration of complete response was 11 months. The most common adverse effects not requiring dose reduction were fever (80%), abdominal pain (32%), and leukopenia (52%) [14]. In a study with 39 patients with ovarian cancer given intraperitoneal IFNα-2b in combination with chemotherapy, 14/35 patients achieved a pathological CR with only one patient discontinuing treatment due to severe fatigue [15]. In a pharmacokinetics study, IP and blood levels of IFNα-2b were measured after administration. All patients had metastatic disease within the peritoneal cavity and were given IFNα-2b in a range of from 5 × 10^6^ to 15 × 10^6^ units. Importantly, the bioavailability of IP IFNα-2b was found to be 30-fold higher intraperitoneally compared to in the peripheral blood, with a slower elimination half-life (10–32 h IP compared to 5–13 h in the peripheral blood) [16].

The first study of high dose IP (range 0.05 × 10^6^/m^2^ IU to 8 × 10^6^/m^2^) IFN-γ for the treatment of ovarian cancer enrolled 27 patients with relapsed, chemo-resistant disease. Unfortunately, none of the patients on this small trial showed objective response, with 14 patients having progressive disease during the protocol [17]. The first IP IFN-γ study enrolled 109 patients with stage III or IV epithelial ovarian cancer. Subjects received IFN-γ either by subcutaneous injection or IP injection through an implanted catheter. Twenty-three patients (21%) had a complete response, while 8 (7%) patients had a partial response [18]. In a study with 7 patients treated with IP IFN-γ, there was an increase in NK cell activity and tumor-associated lymphocytes and macrophages but no objective responses [19]. A similar study with 8 patients treated with IP IFNγ found similar rises in tumor associated lymphocytes and macrophages, 1 patient with a complete response, 2 with partial responses and 2 with stable disease [20]. Pharmacokinetic studies showed that IFN-γ was retained within the peritoneum for longer than 24 h with up to a 150- to 200-fold increase in peak levels above that in plasma. Release into the blood was found only in the higher doses (> 2 × 10^6^ IU/m^2^), with a peak level reached in the serum at 6 h. The peak levels in the peritoneal cavity increased as IFN-γ dose increased, with 30.7 IU/mL measured in the IP location when 0.05 × 10^6^ IU/m^2^ was administered, and up to 1720 IU/mL detected when 8 × 10^6^ IU/m^2^ was administered. These levels persisted in both the blood and peritoneum for up to 24 h [17]. One of the most important observations from the studies was the tolerable side effect profile, and prolonged IP concentrations of IFNs. No published studies report the co-administration of both IFNα and IFNγ IP.

### Intraperitoneal monocytes

The anti-tumor effect of activated monocytes is well characterized. Monocytes are precursors of resident tissue macrophages, and their functions in normal homeostasis include antigen presentation, phagocytosis of apoptotic cells, and tissue development [21]. Monocytes adapt to their microenvironment and differentiate based on cytokine, chemokine and metabolite expression [22]. In the presence of a tumor, monocytes differentiate into classical M1 macrophages that inhibit tumor proliferation and secrete proinflammatory cytokines and chemokines. They are also able to promote natural killer (NK) cell differentiation, providing a rapid cytotoxic response to tumor cells [23, 24]. This response is further potentiated by the release of IFNs by NK cells, promoting the tumoricidal effects of these cells [25]. Macrophages differentiated towards an M1 phenotype can potentially be exploited in the clinic to selectively target tumor cells without damaging normal tissue [26]. Monocytes can also differentiate into M2 (alternative macrophages) that promote tumor proliferation [27–29] and are associated with a poor prognosis in advanced EOC [30]. Therefore, the success of monocytes as an anti-tumor treatment approach may depend on the ability to maintain the M1 phenotype and avoid M2 differentiation in the tumor microenvironment.

Several studies have shown that monocytes can be isolated from the peripheral blood in large numbers (> 1 billion) and at relatively high purity (80–95%) [31, 32]. Two Phase 1 safety trials studied intraperitoneal infusion of autologous monocytes activated with IFNγ or muramyl tripeptide phosphatidylethanolamine (MTP-PE) [33, 34]. These studies showed that IP administration of IFN stimulated monocytes was safe and feasible for the treatment of peritoneal carcinoma of colorectal origin. Indium-111 (^111^In) labeled monocytes were visualized using whole-body Gamma Camera Imaging and found to be confined to the peritoneal cavity and absent from the lungs, heart or liver after 5 days of monitoring [33]. A subsequent study assessed autologous transplantation of monocytes stimulated with liposomal-muramyl tripeptide phosphatidyl ethanolamine (L-MTP-PE), a potent adjuvant, for the treatment of carcinomas of the peritoneum. Patients’ abdomens were imaged at 30 min and 7 days post-infusion. Once again, the ^111^In was confined to the peritoneal cavity with no measurable activity in liver or lungs. Patients experienced low grade toxicities of fevers, chills and abdominal pain [31]. Response rates were not reported.

### Preclinical studies of monocytes combined with both Interferons alpha and gamma

**Fig. 1:**
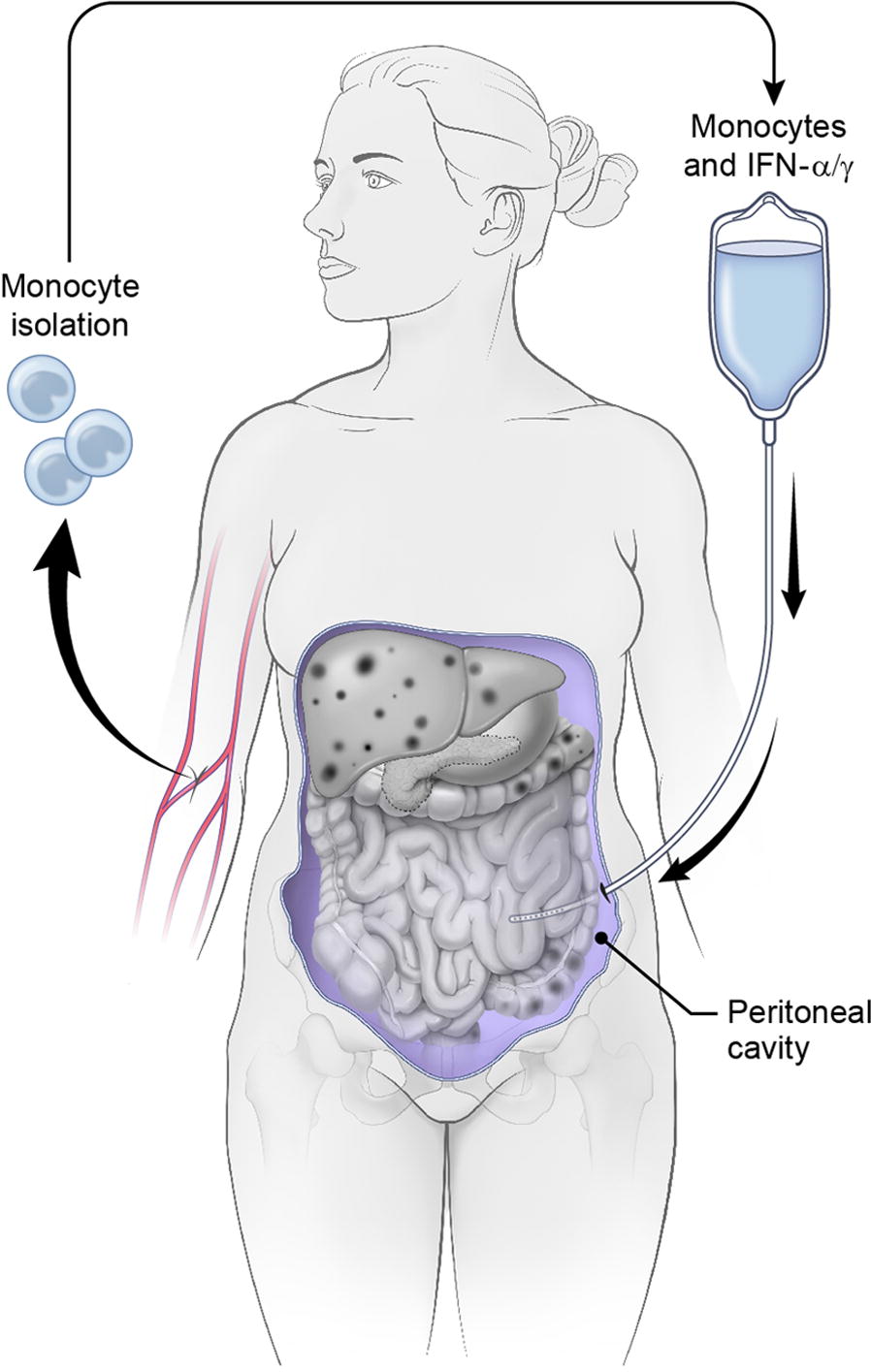
Diagram of treatment. Prior to the start of the trial patients will have an implantable port that access the peritoneum placed surgically. Patients will have their monocytes isolated by counter-flow elutriation and stimulated with Sylatron^®^ (Peginterferon alfa-2b) and Actimmune^®^ (Interferon gamma-1b) ex vivo. The monocytes and interferons will be infused IP by gravity in 250 mL Plasmalyte A, followed by a 250 mL saline wash. The patient will rotate from the supine position to left prostrate, followed by left prostrate, every 15 min for 2 h to ensure movement of the product within the peritoneum

Given the recent successes of immunotherapy in other malignancies, intraperitoneal immunotherapy is a promising area for exploration in cancers predominantly confined to the peritoneal cavity, such as ovarian cancer. Our experimental approach, focused on the innate immune system, is based on previous observations that IFNs themselves are potent anti-neoplastic proteins [35]. We have shown in vitro and in animal models that monocyte stimulation with both IFN-α and IFN-γ achieves M1 differentiation, important for anti-tumor activity. These human monocytes, in combination with human interferons (IFNs) alpha (IFNα-2a or PEGylated IFNα-2b) and gamma (IFNγ-1b) are potent killers of cancer cell lines in vitro [36, 37]. Our studies included 6 ovarian cancer cell lines, and demonstrated that the presence of monocytes additionally decreased viability of some cell lines at low doses of interferons [10]. We have previously published that monocytes treated with both IFNa and IFNg differentiate into the M1 phenotype, and away from the M2 phenotype. In an orthotopic mouse model we showed that intratumoral injection of monocytes and interferons resulted in significant decreases in tumor volume and increase in overall survival [38]. Histology of the tumors showed that the monocytes in the tumor differentiated into inflammatory (M1) macrophages creating an inflammatory environment marked by apoptotic cell death. We also showed that the M1 phenotype persists even in the presence of tumor cells. The persistence of the M1 phenotype was demonstrated in the mouse xenografts, where M1 markers were identified on the cells within the tumor microenvironment. In the same study we also showed that the treatment did not result in toxicity to the liver, kidney, lung or spleen of treated animals [38]. Together, these data provide in vitro and in vivo evidence that monocytes and IFNs result in ovarian cancer cell and tumor death and is a therapeutic approach for the treatment of ovarian cancer (Fig. 1). Based on this evidence, we designed the first clinical trial to test this phenomenon in patients.

### Study design

**Fig. 2:**
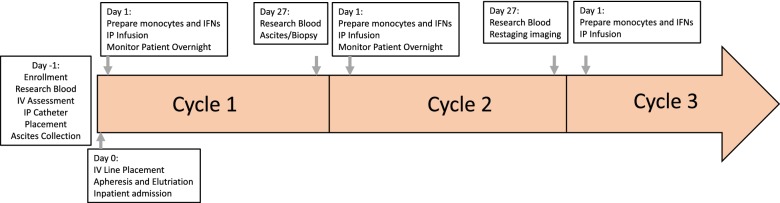
Time line of treatment: After assessment and consent, the patients will be enrolled on Day-1 for line placement and research bloods. On Day 0 the patient will undergo apheresis. The Sylatron^®^ (Peginterferon alfa-2b) and Actimmune^®^ (Interferon gamma-1b) will be added to the monocytes and the product will be infused on Day 1. The patient will be monitored for 24 h and released. This schedule will repeat for Cycle 2. Prior to cycle the patient will have disease re-staging based on a CT scan. If the patient is eligible for cycle three, they will receive the product infusion and be released 3 h post infusion

This is a Phase 1 clinical trial, and the primary endpoint is safety of the regimen. This is a single arm dose escalation study including an expansion cohort at the maximum tolerated dose (MTD) (Table 1). A 3 + 3 Phase I trial of monocytes with Sylatron^®^ (Peginterferon alfa-2b) and Actimmune^®^ (Interferon gamma-1b) dose escalation will proceed to determine the MTD (Table 1). The dose-limiting toxicity (DLT) period is during the first cycle (28 days). For each dose level, three patients are enrolled. There is a 2 week wait from the time of cell infusion from one patient until apheresis and cell infusion of the next patient. If any patient experiences DLT, then three additional patients will be enrolled at that dose level. All patients on a dose level must complete the 28-day cycle prior to beginning enrollment on the next dose level (Fig. 2). If any patient experiences DLT related to experimental agents or study procedures in subsequent cycles, enrollment is placed on hold until evaluated by the IRB and FDA.

**Table 1:** Time line of line placement, monocyte isolation, stimulation with IFNs, and infusion

Dose level	Monocytes total number	SYLATRON^®^ peginterferon alfa2b, mcg	ACTIMMUNE ^®^ interferon gamma-1b, mcg
1	0	25	5
2	75 × 10^6^	25	5
3	750 x 10^6^	25	5
3b	0	250	50
4	750 x 10^6^	250	50

Using a standard 3 + 3 phase 1 clinical design patients in levels 1 and 3b will receive Sylatron^®^ (Peginterferon alfa-2b) and Actimmune^®^ (Interferon gamma-1b) at low dose and high dose respectively

Patients enrolled in levels 2, 3 and 4 will receive escalating doses of monocytes and Sylatron^®^ (Peginterferon alfa-2b) and Actimmune^®^ (Interferon gamma-1b), with monocyte number increasing before Sylatron^®^ (Peginterferon alfa-2b) and Actimmune^®^ (Interferon gamma-1b)

After enrollment, patients have intravenous access scheduled for apheresis in the Department of Transfusion Medicine (DTM), and tunneled IP catheter placed in interventional radiology (Fig. 2). The patient additionally has the option to have an IP port surgically placed. The tunneled IP catheter can be placed prior to start of the protocol. If patient already has existing IP port, this can be used for IP infusion, and a new tunneled catheter will not be placed. Research blood is collected, and ascites is collected if present. In the expansion cohort, solid tumor will be biopsied if amenable at the time of catheter placement and prior to the second infusion. The purpose of the biopsy is to assess monocyte infiltration and differentiation related to treatment.

The patient is admitted to inpatient hospital. On Day 0, the patient undergoes apheresis in the DTM. Monocytes are elutriated, and purity and cell number are assessed. Two-thirds of the monocytes are frozen and stored for future infusion at the DTM in liquid nitrogen. The monocytes are then stored at 4°C overnight. On Day 1 monocytes are warmed to room temperature and the Sylatron^®^ (Peginterferon alfa-2b) and Actimmune^®^ (Interferon gamma-1b) are added at the appropriate concentrations. In order to subvert the immunosuppressive environment of ovarian cancer we are stimulating the patient’s monocytes ex vivo prior to infusion. Excess product is removed to yield a final volume of 250 mL. The excess product will be tested for release criteria, endotoxin, and long term microbial assays. Product cells and supernatant are collected for research analysis of cytokine levels, and monocyte function. After 4 h the product is released and infused within 1 h after the release.

### Criteria for dose escalation

Patients who do not complete treatment in cycle 1 for reasons other than study product-related toxicity will be replaced. If 1 of 3 patients experiences DLT, the dose level will be expanded to 6 patients. If 2 patients in a dose level of 3–6 patients experiences DLT, then the MTD will have been exceeded and the next lower dose level will be expanded to 6 patients. MTD will be the highest dose at which 0–1 of 6 patients experience DLT. If the planned highest dose level is reached and determined to be safe after expansion to 6 patients, it will be considered the MTD. An expansion cohort of 10 additional patients will be treated at the MTD and the results combined with those of patients at the MTD to obtain improved estimates of safety and toxicity as well as to perform analyses to address secondary objectives.

### Primary outcome measurements

#### Adverse events

All patients who signed consent will be evaluable for toxicity. Expected adverse reactions of Sylatron^®^ (Peginterferon alfa-2b) when given subcutaneously include depression and other neuropsychiatric disorders as a black box warning. Additional adverse reactions (> 60%) include fatigue, increased AST and ALT, pyrexia, anorexia, myalgia, nausea. Contraindications include a known hypersensitivity reaction to Peginterferon alfa-2b or interferon alfa-2b, autoimmune hepatitis and hepatic decompensation with Child–Pugh score class B and C. Actimmune is metabolized by cytochrome P-450 (CYP) enzymes, and we will monitor closely when used in combination with drugs metabolized by CYP2C9 or CYP2D6 [39].

Expected adverse reactions of Actimmune^®^ (Interferon gamma-1b) include AST/ALT elevation, exacerbation of a previous cardiac condition, reversible neutropenia and thrombocytopenia, gait disturbance, seizure. Other adverse effects include pyrexia, headache, rash, chills, injection site erythema or tenderness, fatigue, diarrhea, vomiting, nausea, myalgia and arthralgia. Contraindications include patients who develop or have known hypersensitivity to interferon-gamma, *E. coli* derived products, or any component of the product. Caution is needed when administering Actimmune in combination with other potentially myelosuppressive agents. Actimmune may decrease cytochrome P-450 concentrations. Actimmune^®^ (Interferon gamma-1b) [40].

Both Sylatron^®^ (Peginterferon alfa-2b) and Actimmune^®^ (Interferon gamma-1b) can cause flu-like symptoms. The addition of both Sylatron^®^ (Peginterferon alfa-2b) and Actimmune^®^ (Interferon gamma-1b) and pro-inflammatory monocytes can result in symptoms that look similar to a peritoneal infection. To address the possibility of these symptoms overlying or mimicking an active infection, the patient will start a course of broad spectrum antibiotics after the catheter and any fluid collected near the tip of the catheter will be sent to be cultured in the clinical laboratory.

### Secondary outcome measurements

#### Response criteria

For the purposes of this study, patients are re-evaluated for response every 8 weeks. In addition to a baseline scan, confirmatory scans should also be obtained 4–8 (not less than 4) weeks following initial documentation of objective response. Response and progression are evaluated in this study using the international criteria proposed by the revised Response Evaluation Criteria in Solid Tumors (RECIST) guideline (version 1.1) [41]. Changes in the largest diameter (unidimensional measurement) of the tumor lesions and the shortest diameter in the case of malignant lymph nodes are used.

Only those patients who have measurable disease present at baseline, have received at least one cycle of therapy, and have had their disease re-evaluated will be considered evaluable for response. Patients are not stratified by histology since all have received multiple prior regimens, and there is no evidence to suggest that histology would influence response to treatment at this stage of disease recurrence. These patients will have their response classified according to the definitions stated below. Furthermore, patients who exhibit objective disease progression prior to the end of cycle 1 will also be considered evaluable. Patients who have lesions present at baseline that are evaluable but do not meet the definitions of measurable disease, have received at least one cycle of therapy, and have had their disease re-evaluated will be considered evaluable for non-target disease. The response assessment is based on the presence, absence, or unequivocal progression of the lesions.

### Tumor markers

Tumor markers alone cannot be used to assess response. If markers are initially above the upper normal limit, they must normalize for a patient to be considered in complete clinical response. Specific guidelines for both CA-125 response (in recurrent ovarian cancer) and PSA response (in recurrent prostate cancer) have been published [42–44]. In addition, the Gynecologic Cancer Intergroup has developed CA-125 progression criteria which are to be integrated with objective tumor assessment for use in first-line trials in ovarian cancer [45].

### Pharmacodynamic measurements

We have designed a series of prospectively collected secondary measurements to help understand the physiological response, and generate potential correlates of efficacy or failure. All patients will have peripheral blood collected before and after each treatment. peripheral blood mononuclear cells (PBMC) and plasma will be collected and stored in liquid nitrogen or at − 80°C respectively. At the end of the study plasma will be used to measure for the presence of anti-interferon alpha and anti-interferon gamma antibodies. Furthermore, using multiplex, 42-plex Human panel, we will measure the presence of 42 cytokines and proteins for correlation studies with patient response. (see statistical justification below). PBMC will be analyzed using multi-panel, multi-parameter flow cytometry to measure 127 immune cell subsets for correlation with patient response. The PBMC and multiplex arrays may also provide insight into whether heavily pre-treated patients have a unique immune signature.

### Statistical considerations

Patients will be accrued in standard 3 + 3 dose escalation design, with expansion to 6 patients at the MTD and 10 additional patients at the MTD to allow for sufficient patients to perform correlative studies. The 16 patients treated at the MTD (6 from dose escalation/MTD determination plus 10 additional patients) will be used to evaluate biologic correlates. With 16 patients, there would be 80% power to detect a change (either actual change or fold/relative change as appropriate) in the number of activated monocytes from baseline to after one cycle of treatment which would be equal to ¾ of a standard deviation of the difference measured (effect size 0.75) with a two-tailed 0.05 significance level paired *t* test. If there are usable paired results from 10 patients instead of 16, this would result in the ability to detect a change with a 1.0 effect size based on the same parameters. In addition, a large number of other parameters will be obtained via flow cytometry and the changes from baseline to after one cycle of therapy will be determined. Given the large number of possible tests performed and the exploratory nature of the evaluation, there will not be any formal adjustment for multiple comparisons for these parameters. However, with 16 patients, there would be 80% power for any of the tests comparing baseline to post-treatment to detect a one SD difference between the two time points (effect size 1.0) with a two-tailed 0.01 significance level paired t-test; if there are 10 patients, there would be 80% power to detect a 1.0 effect size with a two-tailed 0.05 significance level paired t-test. The results of these exploratory tests will be reported in the context of the number of such tests performed. The clinical response rate (in patients with measurable disease) and time to progression (in all patients) will also be estimated in a preliminary fashion. Appropriate confidence intervals will be provided along with fractions responding and a Kaplan–Meier curve of TTP. It is estimated that up to 24 patients will be required to complete all 4 dose levels plus 10 additional patients will be needed at the MTD. Thus, up to 34 evaluable patients may be required. In order to allow for a small number of inevaluable patients, the accrual ceiling will be set at 40 patients. Expected accrual rate is 1–2 patients per month.

### Innovation

We have developed a novel immunotherapy focused on the innate immune system for the treatment of ovarian cancer. We have combined the use of autologous monocytes and interferons alpha and gamma for local–regional administration directly into the peritoneal cavity. This therapy is highly unique in that it only uses components of the innate immune response. IFNs have been shown to be both cytostatic and cytotoxic to cancer cells. Despite early promising results using IFN-α IP for patients with ovarian cancer, there were few follow up studies. The toxicities associated with very high dose IFN-α and the contemporary discovery and use of novel chemotherapeutics obscured the development of IFNs as anti-cancer agents in patients with ovarian cancer. It has been known for some time that IFNs induce a systemic pro-inflammatory response. Malignant cancer, however, promotes an anti-inflammatory environment. The goal of this clinical trial is to modulate the innate immune system in order to reverse immune suppression and promote anti-tumor immunity. The anti-tumor effect of M1 polarized monocytes can potentially be exploited in the clinic to selectively target tumor cells, but the success of monocytes as an anti-tumor treatment approach will rely on maintaining the M1 phenotype and avoiding M2 differentiation in the tumor microenvironment. Combining Sylatron^®^ (Peginterferon alfa-2b) and Actimmune^®^ (Interferon gamma-1b) and autologous monocytes with monocytes is a novel approach to maximize the anti-tumor properties of all three agents.

## Discussion

This is the first clinical trial to combine three agents, Sylatron^®^ (Peginterferon alfa-2b) and Actimmune^®^ (Interferon gamma-1b) and autologous monocytes, as an intraperitoneal infusion for women with recurrent platinum-refractory ovarian cancer. This novel immunotherapy focused uniquely on the innate immune system for the treatment of ovarian cancer. Enrollment began in January 2017 and the estimated date for completion of primary endpoint is January 2019. Results from this trial will determine the MTD of the three agents. In addition, we will gain information on the feasibility of this route of administration, and the processing and storing of the cellular product prior to infusion. Exploratory correlative studies will generate new hypotheses for the mechanism of monocyte anti-tumor activity, which may shed light on how to optimize this regimen in the context of standard chemotherapy or novel anti-cancer agents.

Adoptive cell therapy has largely failed in the treatment of solid tumors, despite the early success of T cell therapy for the treatment for blood malignancies, and the limited success of dendritic cell based vaccines. An immunosuppressive tumor environment may inhibit or block adoptive T cell therapy. Metastatic ovarian cancer has many immunosuppressive properties. Both the presence of inhibitory molecules in the malignant ascites, and the presence of regulatory T cells in the tumor suggest that one of the primary functions of the cancer is to suppress and modify the immune response. These observations fit the detection, equilibrium and evasion mechanism of immune–tumor interactions.

In the context of ovarian cancer, the late diagnosis of advanced disease, the generation of an immunosuppressive environment, and the unusual anatomy of peritoneal carcinomatosis make the application of immune cell therapies especially difficult. The pattern of vascularization in the malignant peritoneal fluid greatly limits the ability to deliver agents to the malignant tissue. The clinical observations that using IP chemotherapy or HIPEC increases overall survival strongly indicate that locoregional therapy is superior to parenteral therapy in this clinical scenario. Thus, it is logical that cellular products would similarly reach their target more efficiently when administered IP.
